# Supplementary material for: Identification of a Novel Antibacterial Function of Mammalian Calreticulin
Source: Biomolecules. 2025 Jul 4;15(7):966. doi: 10.3390/biom15070966 (PMC12292289; doi:10.3390/biom15070966)
Supplement: Supplementary file 1 [file biomolecules-15-00966-s001.zip › biomolecules-3722123-supplementary.pdf]

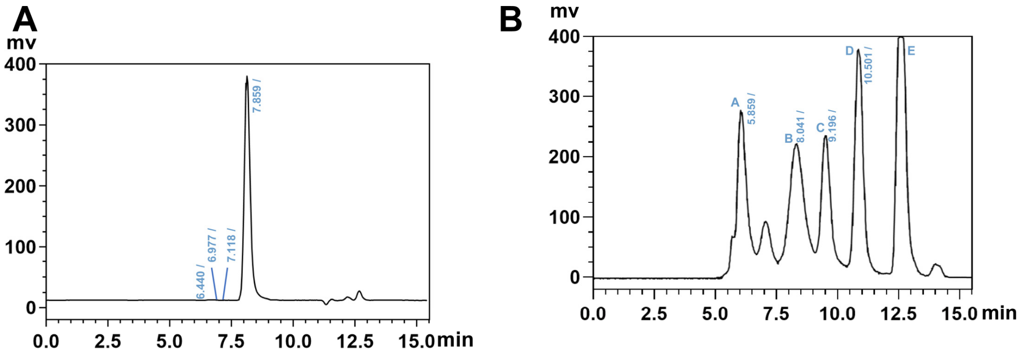

	Component	Molecular Weight(Da)
A	Thyroglobulin(bovine)	670,000
B	G-globulin(bovine)	158,000
C	Ovalbumin(chicken)	44,000
D	Myoglobin(horse)	17,000
E	Vitamin B12	1,350

Figure S1. (**A**) SEC-HPLC analysis of the molecular weight and purity of recombinant human calreticulin. (**B**) SEC-HPLC analysis of standards.

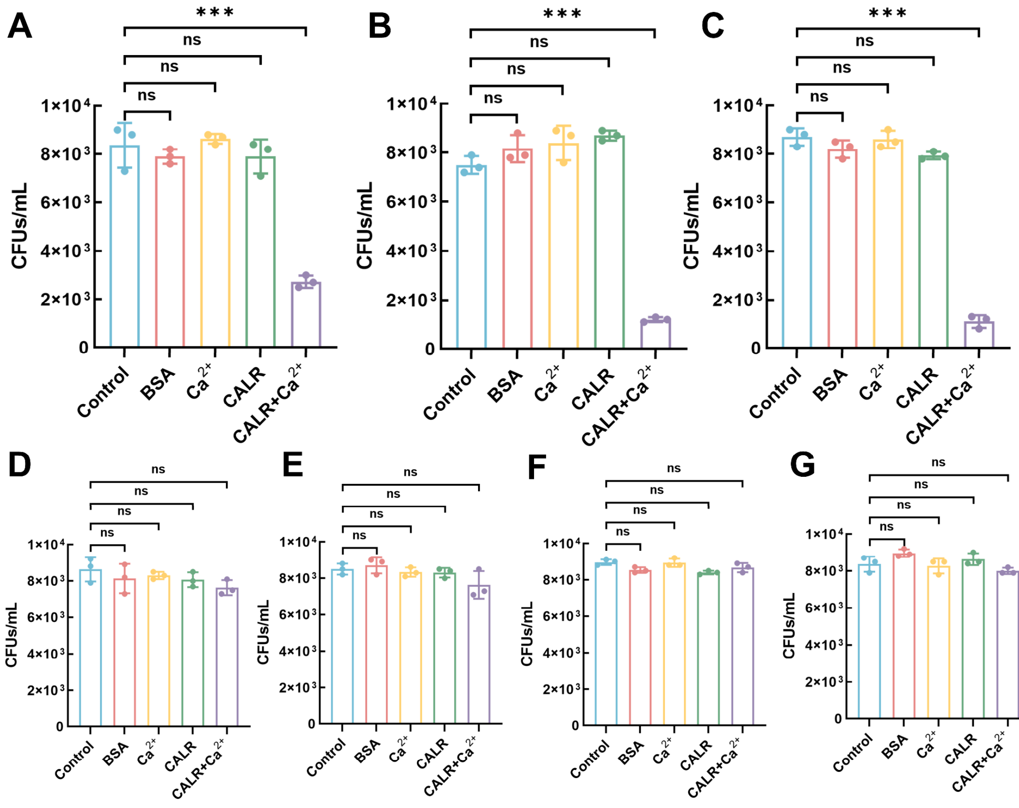

Figure S2. (**A**-**C**) CFU assays were conducted to assess the inhibitory effects of recombinant human calreticulin on *Escherichia coli*, *Salmonella typhimurium* and *Pasteurella multocida*. (**D**, **E)** CFU assays evaluating the antibacterial activity of recombinant goat calreticulin against *Streptococcus* and *Staphylococcus aureus*. (**F**, **G**) CFU assays were performed to evaluate the inhibitory effects of recombinant human calreticulin against *Streptococcus* and *Staphylococcus aureus*. All data shown are the mean ± SD from three independent experiments. Statistical significance was obtained using one-way ANOVA. ns, no significance; ****P* < 0.001.

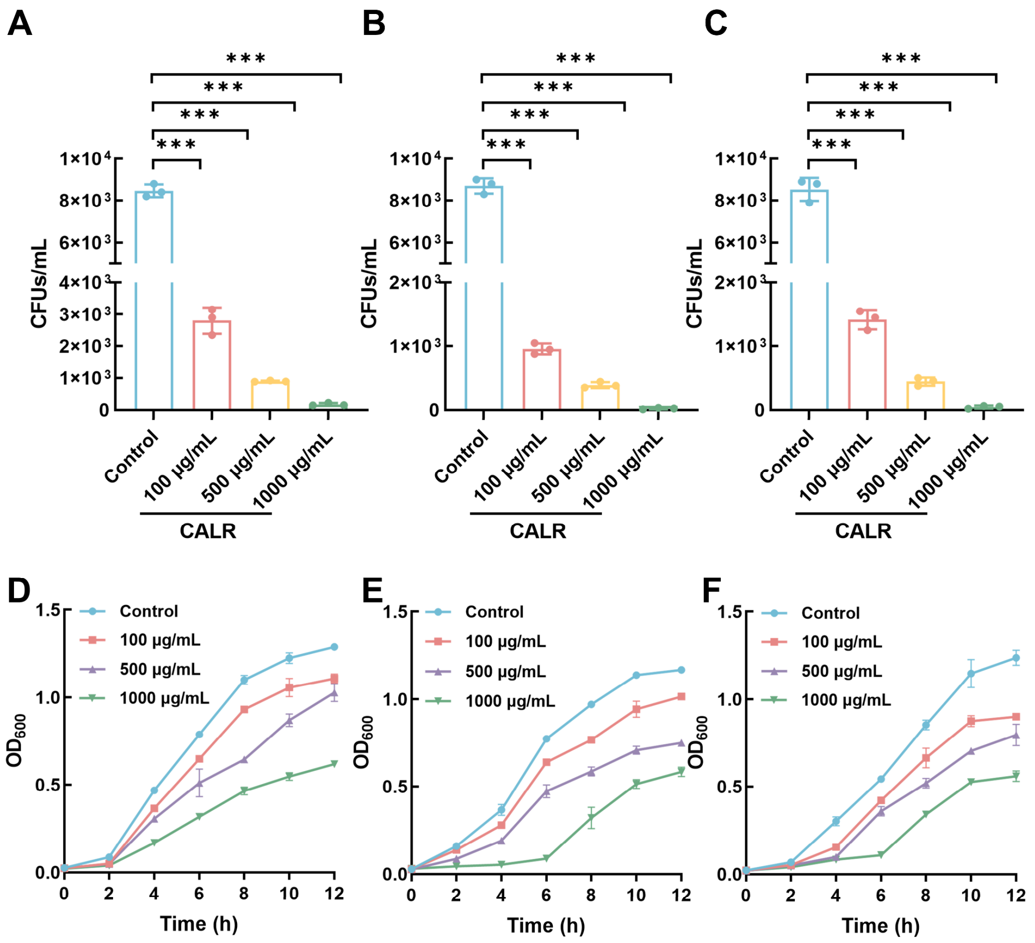

Figure S3. Calreticulin inhibits bacterial growth in a concentration-dependent manner. (**A**-**C**) *Escherichia coli*, *Salmonella typhimurium*, and *Pasteurella multocida* were incubated with increasing concentrations of calreticulin (100 μg/mL, 500 μg/mL, and 1000 μg/mL) in the presence of 10 mM Ca^{2+}. The control group was treated with buffer only. (**D**-**F**) Growth curves of *Escherichia coli*, *Salmonella typhimurium*, and *Pasteurella multocida* in the presence of different concentrations of calreticulin (100 μg/mL, 500 μg/mL, and 1000 μg/mL) were measured. Buffer-treated bacteria served as the control group. Statistical significance was obtained using one-way ANOVA. ***$P < 0.001$.

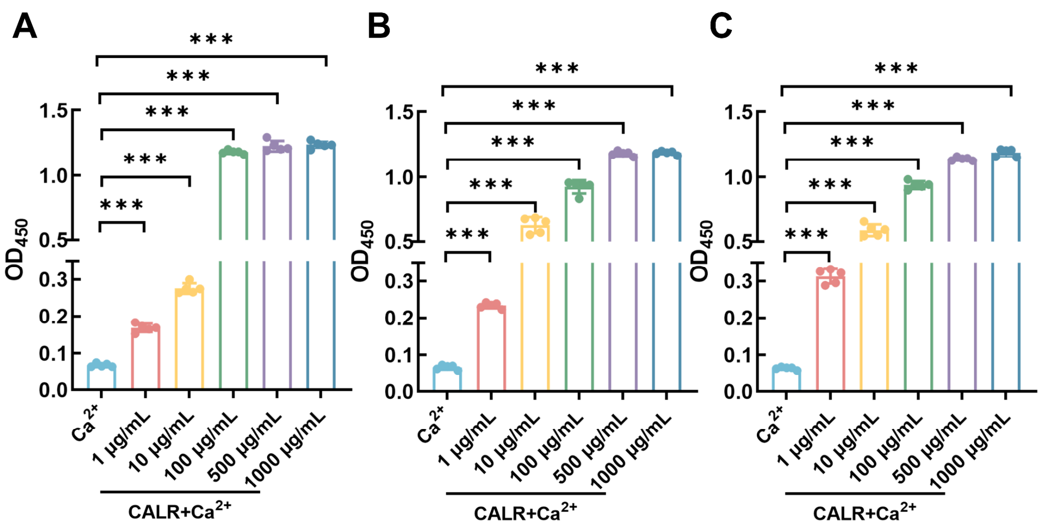

Figure S4. Microorganism-binding activity of calreticulin. (**A**-**C**) ELISA was performed to assess the binding of calreticulin at various concentrations (1 μg/mL, 10 μg/mL, 100 μg/mL, 500 μg/mL, and 1000 μg/mL) to immobilized *Escherichia coli*, *Salmonella typhimurium*, and *Pasteurella multocida* in the presence of 10 mM Ca^{2+}. Statistical significance was obtained using one-way ANOVA. ***$P < 0.001$.

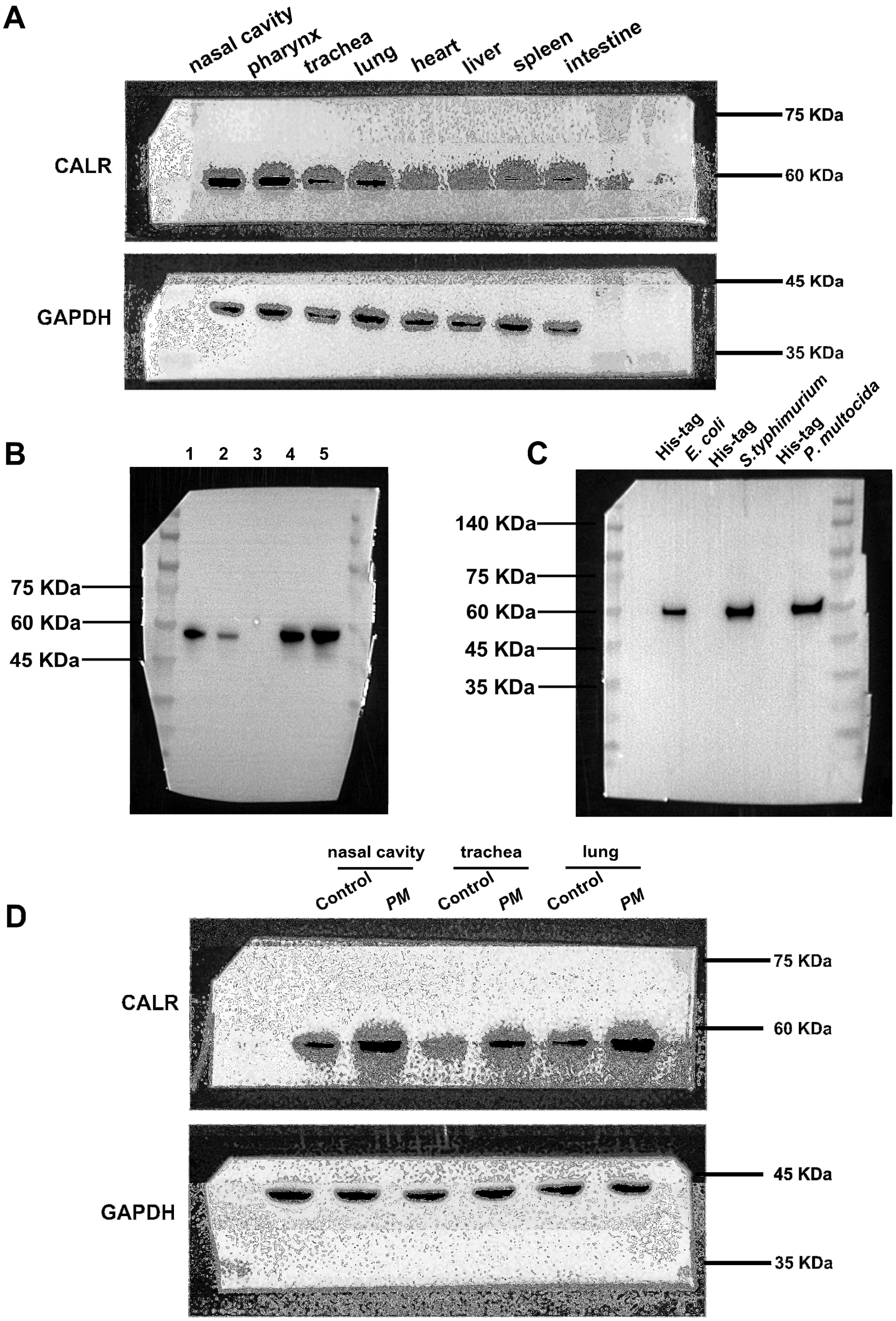

Figure S5. Original Western blot images. (**A**) Original blot images for Figure 2B. (**B**) Original blot images for Figure 3D. (**C**) Original blot images for Figure 5D. (**D**) Original blot images for Figure 6D.

Table S1. Primer sequences used for RT-qPCR.

Gene	Primers sequence (5′-3′)	Orientation
CALR	ATTCGCTGCAAGGACGATGA	Forward
	GGCACGATCATCCCAGTCTT	Reverse
GAPDH	TTATGACCACTGTCCACGCC	Forward
	TCAGATCCACAACGGACACG	Reverse
